# Evaluation of Changes in Veterans Affairs Medical Centers’ Mortality Rates After Risk Adjustment for Socioeconomic Status

**DOI:** 10.1001/jamanetworkopen.2020.24345

**Published:** 2020-12-03

**Authors:** Amal N. Trivedi, Lan Jiang, Gabriella Silva, Wen-Chih Wu, Vincent Mor, Michael J. Fine, Nancy R. Kressin, Roee Gutman

**Affiliations:** 1 Center of Innovation for Long-term Services and Supports, Providence Veterans Affairs Medical Center, Providence, Rhode Island; 2 Department of Health Services, Policy and Practice, Brown University School of Public Health, Providence, Rhode Island; 3 Department of Biostatistics, Brown University School of Public Health, Providence, Rhode Island; 4 Center for Health Equity Research and Promotion, Veterans Affairs Pittsburgh Healthcare System, Pittsburgh, Pennsylvania; 5 Division of General Internal Medicine, School of Medicine, University of Pittsburgh Medical Center, Pittsburgh, Pennsylvania; 6 Center for Healthcare Organization and Implementation Research, Veterans Affairs Boston Healthcare System, Boston, Massachusetts; 7 Division of General Internal Medicine, Boston University School of Medicine, Boston, Massachusetts

## Abstract

**Importance:**

Socioeconomic factors are associated with worse outcomes after hospitalization, but neither the Centers for Medicare & Medicaid Services (CMS) nor the Veterans Affairs (VA) health care system adjust for socioeconomic factors in profiling hospital mortality.

**Objective:**

To evaluate changes in Veterans Affairs medical centers’ (VAMCs’) risk-standardized mortality rates among veterans hospitalized for heart failure and pneumonia after adjusting for socioeconomic factors.

**Design, Setting, and Participants:**

In this cross-sectional study, retrospective data were used to assess 131 VAMCs’ risk-standardized 30-day mortality rates with or without adjustment for socioeconomic covariates. The study population included 42 892 veterans hospitalized with heart failure and 39 062 veterans hospitalized with pneumonia from January 1, 2012, to December 31, 2014. Data were analyzed from March 1, 2019, to April 1, 2020.

**Main Outcomes and Measures:**

The primary outcome was 30-day mortality after admission. Socioeconomic covariates included neighborhood disadvantage, race/ethnicity, homelessness, rurality, nursing home residence, reason for Medicare eligibility, Medicaid and Medicare dual eligibility, and VA priority.

**Results:**

The study population included 42 892 veterans hospitalized with heart failure (98.2% male; mean [SD] age, 71.9 [11.4] years) and 39 062 veterans hospitalized with pneumonia (96.8% male; mean [SD] age, 71.0 [12.4] years). The addition of socioeconomic factors to the CMS models modestly increased the C statistic from 0.77 (95% CI, 0.77-0.78) to 0.78 (95% CI, 0.78-0.78) for 30-day mortality after heart failure and from 0.73 (95% CI, 0.72-0.73) to 0.74 (95% CI, 0.73-0.74) for 30-day mortality after pneumonia. Mortality rates were highly correlated (Spearman correlations of ≥0.98) in models that included or did not include socioeconomic factors. With the use of the CMS model for heart failure, VAMCs in the lowest quintile had a mean (SD) mortality rate of 6.0% (0.4%), those in the middle 3 quintiles had a mean (SD) mortality rate of 7.2% (0.4%), and those in the highest quintile had a mean (SD) mortality rate of 8.8% (0.6%). After the inclusion of socioeconomic covariates, the adjusted mean (SD) mortality was 6.1% (0.4%) for hospitals in the lowest quintile, 7.2% (0.4%) for those in the middle 3 quintiles, and 8.6% (0.5%) for those in the highest quintile. The mean absolute change in rank after socioeconomic adjustment was 3.0 ranking positions (interquartile range, 1.0-4.0) among hospitals in the highest quintile of mortality after heart failure and 4.4 ranking positions (interquartile range, 1.0-6.0) among VAMCs in the lowest quintile. Similar findings were observed for mortality rankings in pneumonia and after inclusion of clinical covariates.

**Conclusions and Relevance:**

This study suggests that adjustments for socioeconomic factors did not meaningfully change VAMCs’ risk-adjusted 30-day mortality rates for veterans hospitalized for heart failure and pneumonia. The implications of such adjustments should be examined for other quality measures and health systems.

## Key Points

**Question:**

Does risk adjustment for socioeconomic status change Veterans Affairs Medical Centers’ rankings for mortality rates after hospitalization for heart failure and pneumonia?

**Findings:**

In this cross-sectional analysis, Veterans Affairs medical centers’ mortality rates were highly correlated in models that included or did not include socioeconomic factors, and few medical centers’ rates were associated with substantial changes in risk-adjusted mortality after adjustment for socioeconomic status.

**Meaning:**

This study’s finding suggest that Veterans Affairs medical centers’ performance on mortality measures after hospitalization for heart failure and pneumonia were minimally changed after risk adjustment for socioeconomic factors.

## Teaser

This cross-sectional study evaluates changes in Veterans Affairs (VA) medical centers’ risk-standardized mortality rates among veterans hospitalized for heart failure and pneumonia after adjusting for socioeconomic factors.

## Introduction

Nearly all hospitals in the United States, including all Veterans Affairs medical centers (VAMCs), report mortality rates for patients hospitalized for common medical and surgical conditions.^1^ Performance on these outcome measures carries high stakes. The Centers for Medicare & Medicaid Services (CMS) adjust payments to hospitals based on their performance on quality-of-care measures, including hospital mortality rates after acute myocardial infarction, heart failure (HF), and pneumonia.^2^ Hospital mortality rates inform the Veterans Affairs (VA) Strategic Analytic and Information Learning ratings, which the VA health system uses to measure performance in VAMCs.^3^ The Veterans Access, Choice, and Accountability Act of 2014 requires the VA health system to release comprehensive data to the public on access to, quality of, and outcomes of care, including hospital mortality.

Valid hospital outcome measures must adequately account for differences in patients’ risk.^1^ Much of the prior concern with risk adjustment has involved the source of the data (administrative vs clinical records), the selection of appropriate covariates, or the optimal approach to statistical modeling.^4,5,6^ Fewer studies have examined the role of socioeconomic status (SES) and other social risk factors in risk adjustment, although these factors are associated with worse postdischarge outcomes and vary markedly across private sector hospitals.^7,8,9^ Furthermore, prior studies have documented concerns about the validity of claims-based measures of clinical risk.^10,11^ Socioeconomic factors may capture heterogeneity in clinical risk that is not correlated with claims-based measures of severity. Despite emerging consensus from stakeholder groups that profiling performance should include consideration of patients’ sociodemographic characteristics, neither CMS nor the VA health system include sociodemographic factors other than age and sex in risk-adjusted models of hospital mortality.^9,12,13,14,15^ The 21st Century Cures Act requires CMS to adjust financial penalties (but not risk-standardized readmission rates) based on the hospitals’ proportion of patients with dual Medicaid and Medicare eligibility.^16^

Prior studies have found conflicting evidence on the association of socioeconomic risk adjustment with hospital rankings.^16,17,18,19,20,21^ These studies have focused exclusively on readmissions and most have used claims-based conditions, rather than objective clinical data, to account for severity of illness. The association of adjustment for socioeconomic factors with assessments of hospital mortality is not well understood.

The objective of this study was to develop and test risk-adjustment approaches that incorporate veterans’ sociodemographic characteristics (neighborhood disadvantage, income, Medicaid enrollment, homelessness, race/ethnicity, and nursing home residence) into assessments of hospital mortality. We conducted a retrospective, observational, cross-sectional study that tested the performance of models that did or did not include sociodemographic characteristics and assessed the association of the inclusion of sociodemographic characteristics and objective clinical covariates with the rankings of VAMCs for hospital mortality after HF and pneumonia.

## Methods

### Data Sources and Study Population

We merged data from the VA health system’s Corporate Data Warehouse with the CMS Medicare Master Beneficiary Summary File, the Minimum Data Set, and the US Census. The study population included all veterans admitted to a VAMC with a principal discharge *International Classification of Diseases, Ninth Revision, Clinical Modification* (*ICD-9-CM*) diagnostic code of HF or pneumonia from January 1, 2009, to December 31, 2014 (eTable 1 in the Supplement). We used hospitalizations in calendar years 2009 to 2011 (based on admission date) to derive the risk-adjustment model. The primary study population was individuals hospitalized in calendar years 2012 to 2014, which we used to report model discrimination and hospital-level risk-adjusted mortality. We randomly sampled 1 hospitalization for veterans with more than 1 admission from 2012 to 2014.^22,23,24^ This study followed the Strengthening the Reporting of Observational Studies in Epidemiology (STROBE) reporting guideline for cross-sectional studies. The Providence VAMC Institutional Review Board approved the study protocol and waived the need for informed consent because the risk to the study participants was no more than minimal and the research could not practicably be conducted without the waiver.

Consistent with VA health system and CMS specifications, we excluded hospitalizations from veterans with less than 12 months of continuous VA health system enrollment in the year prior to the admission, those discharged alive within 24 hours, those who left against medical advice, and those with hospice use within 1 year prior to the date of admission (eFigure 1 in the Supplement). For patients who were transferred between VAMCs or from a VAMC to a private sector hospital, the outcome was associated with the hospital in which the veteran was initially admitted.^22,23,24^

### Measures

The primary outcome variable was death from any cause within 30 days after hospital admission, as derived from the VA health system vital status file.^25^ Deaths could occur in the hospital or after discharge.

Covariates from the CMS and VA health system model included age, sex, and claims-based comorbid conditions (22 for HF and 30 for pneumonia) derived from the index admission, as well as outpatient and inpatient encounter data for the 12 months prior to admission (eTable 2 in the Supplement). We did not include comorbid conditions from Medicare claims, consistent with the VA health system’s methods.^26^ Objective clinical variables were selected based on prior literature, clinical face validity, availability at the time of presentation, and the feasibility of routine data extraction.^10^ These variables included systolic blood pressure, diastolic blood pressure, heart rate, respiratory rate, oxygen saturation, body mass index, hematocrit, serum sodium level, serum potassium level, blood urea nitrogen level, serum creatinine level, serum glucose level (pneumonia only), B-type natriuretic peptide level (HF only), and ejection fraction (HF only). Each variable was derived during the 2 days prior to admission until 1 day after admission using the value closest to the admission time.^10^ We used natural language processing methods to extract ejection fraction for patients with HF.^27^ Rates of missing values are shown in eTable 3 in the Supplement.

We included 9 sociodemographic covariates: the Area Deprivation Index (ADI), race/ethnicity, veteran’s priority group, Medicaid enrollment, original reason for Medicare eligibility, homelessness, prior nursing home use in the year before admission, direct admission from a nursing home, and rural residence. Medicaid enrollment and original reason for Medicare enrollment were not available for persons without Medicare coverage, and were therefore limited to analyses of veterans aged 66 years or older.

The ADI is a factor-based composite score of 17 poverty, education, housing, and employment variables derived from the US Census that can be aggregated to the census block or census tract.^28,29^ The measure is constructed with a mean of 100 and standard deviation of 10 nationally. Because it is a relative measure of disadvantage, we assigned each veteran to deciles of the ADI. We used veterans’ census tracts (available in VA Planning Systems Support Group data) to derive their ADI score. For veterans with missing census tracts, we used 9-digit zip codes to link to census block group. If the 9-digit zip code was missing, we used veterans’ 5-digit zip codes. In the validation cohort, the source of the ADI was as follows: 81% from the census tract, 15% from the 9-digit zip code, and 3% from the 5-digit zip code; 1% were missing (eTable 3 in the Supplement). For homeless veterans and nursing home residents, we derived the ADI using the last recorded address in VA health system enrollment records.

The VA health system assigns enrollment priority group on the basis of the presence and degree of service-related disability, income, features of military service, eligibility for Medicaid coverage, and receipt of VA pension benefits, among other factors. For nursing home residence, we constructed a 3-level variable of short-term or long-term nursing home residence on the basis of whether the veteran had no evidence of nursing home use, was in a nursing home for less than 90 days, or was in a nursing home for greater than 90 days in the 1 year prior to the index admission. We also included a binary indicator of whether the hospital admission occurred directly from a nursing home. Although enrollment priority and nursing home residence can reflect health status, we classified them as socioeconomic factors because enrollment priority includes measures of income, assets, and Medicaid eligibility, and nursing home residence reflects patients’ living conditions. Rurality was captured using a rural-urban classification system developed by the VA Planning Systems Support Group in collaboration with the US Census Bureau. Homelessness was defined by the presence of an *ICD-9-CM* code of V60.0 or V.60.1.^30^ Last, we included race/ethnicity (non-Hispanic White, non-Hispanic Black, Hispanic, and other).^31^

### Statistical Analysis

Statistical analysis was performed from March 1, 2019, to April 1, 2020. We constructed hierarchical logistic regression models to estimate 30-day mortality after including the CMS and VA health system claims-based, clinical, and socioeconomic covariates. The models include adjustments for facility-specific intercepts using the hierarchical structure to account for clustering of patients and differential sample sizes within hospitals, consistent with the approach used by the CMS and VA health system. We imputed missing continuous covariates using Markov chain Monte Carlo single imputation. For observations with missing ADI, we imputed the continuous value before assigning the observation to a decile. Observations with missing race/ethnicity (n = 222 [0.5%] for HF; n = 250 [0.6%] for pneumonia) were dropped. The discrimination of each model was reported using the area under the receiver operating characteristic curve (C statistic).

Using the Spearman correlation, we tested the agreement in hospital rankings from models that included or did not include socioeconomic covariates. We estimated the number of hospitals that changed their ranking by more than 10% (ie, 13 ranking positions) and that moved across a quintile threshold after the inclusion of socioeconomic covariates. We report stratified analyses of the association of socioeconomic adjustment for VA hospitals in the highest, lowest, and middle 3 quintiles of risk-standardized mortality (as derived from the CMS and VA health system claims-based model). Last, we repeated all analyses after restricting to patients 66 years or older with Medicare coverage. All analyses were conducted in SAS, version 9.4 (SAS Institute Inc).

## Results

### Characteristics of the Study Population

Among 42 892 veterans hospitalized with HF in 131 medical centers from 2012 to 2014, the mean (SD) age was 71.9 (11.4) years, 98.2% were male, 23.3% were Black, the mean (SD) number of comorbid conditions was 5.9 (3.0), 13.5% received nursing home care prior to admission, 11.2% had evidence of homelessness, and the mean (SD) ADI was 105.2 (13.8) (Table 1). Among 39 062 veterans hospitalized with pneumonia in 131 medical centers from 2012 to 2014, the mean (SD) age was 71.0 (12.4) years, 96.8% were male, 15.6% were Black, the mean (SD) number of comorbid conditions was 5.1 (3.1), 13.7% received nursing home care prior to admission, 12.6% had evidence of homelessness, and the mean (SD) ADI was 105.4 (13.4). The unadjusted 30-day mortality rate was 7.2% for HF and 8.1% for pneumonia; 64.7% (HF) and 56.6% (pneumonia) of deaths occurred after discharge. The prevalence of claims-based comorbidities is shown in eTable 2 in the Supplement. At the hospital level, the interquartile ranges (IQRs) of the proportion of Black patients were 4.3% to 26.9% for HF and 3.6% to 20.3% for pneumonia (eTable 4 in the Supplement). The IQRs for the proportion of rural patients were 19.0% to 56.6% (HF) and 17.0% to 55.7% (pneumonia), and for the proportion of homeless individuals, they were 6.0% to 13.3% (HF) and 7.5% to 16.1% (pneumonia). The IQRs for hospital-level mean ADI score were 102.5 to 110.5 (HF) and 102.2 to 110.1 (pneumonia). eTable 5 in the Supplement shows unadjusted 30-day mortality rates by socioeconomic characteristics.

**Table 1.** Characteristics of Veterans Admitted With Heart Failure and Pneumonia, 2012-2014

Characteristic	Heart failure (n = 42 892)	Pneumonia (n = 39 062)
Age, mean (SD), y	71.9 (11.4)	71.0 (12.4)
Male, No. (%)	42 098 (98.2)	37 796 (96.8)
No. of claims-based comorbidities, mean (SD)	5.9 (3.0)	5.1 (3.1)
Blood pressure, mean (SD), mm Hg		
Systolic	136.1 (24.2)	129.2 (22.9)
Diastolic	77.0 (14.6)	71.4 (13.1)
Heart rate, mean (SD), beats/min	81.2 (16.9)	88.0 (16.8)
Respiratory rate, mean (SD), breaths/min	19.5 (2.8)	19.9 (3.2)
Pulse oximetry, mean (SD), %^a^	95.8 (2.9)	95.0 (3.0)
BMI, mean (SD)	31.3 (7.9)	27.3 (6.8)
Sodium, mean (SD), mEq/L^a^	138.4 (4.3)	136.6 (4.6)
Potassium, mean (SD), mEq/L^a^	4.2 (0.6)	4.1 (0.5)
Blood urea nitrogen, mean (SD), mg/dL	28.5 (16.2)	22.8 (13.1)
Creatinine, mean (SD), mg/dL^a^	1.5 (0.8)	1.4 (0.9)
Hematocrit, mean (SD), %^a^	36.8 (6.3)	36.8 (6.1)
Race/ethnicity, No. (%)^a^		
White	29 540 (68.9)	29 658 (75.9)
Black	9980 (23.3)	6073 (15.6)
Hispanic	2259 (5.3)	2216 (5.7)
Other	891 (2.1)	866 (2.2)
Enrollment priority, No. (%)^b^		
Group 1	10 758 (25.1)	10 765 (27.6)
Group 2	2393 (5.6)	2335 (6.0)
Group 3	3925 (9.2)	3687 (9.4)
Group 4	2212 (5.2)	2355 (6.0)
Group 5	21 189 (49.4)	17 641 (45.2)
Group 6	307 (0.7)	370 (1.0)
Group 7	1988 (4.6)	1783 (4.6)
Group 8	120 (0.3)	126 (0.3)
Nursing home use in year prior to admission, No. (%)	5782 (13.5)	5346 (13.7)
Direct admission from nursing home, No. (%)	988 (2.3)	1547 (4.0)
Rural, No. (%)	14 025 (32.7)	13 992 (35.8)
Area deprivation index, mean (SD)	105.2 (13.8)	105.4 (13.4)
Homeless, No. (%)	4790 (11.2)	4935 (12.6)

Abbreviations: BMI, body mass index (calculated as weight in kilograms divided by height in meters squared); VA, Veterans Affairs.

SI conversion factors: To convert sodium to millimoles per liter, multiply by 1.0; potassium to millimoles per liter, multiply by 1.0; blood urea nitrogen to millimoles per liter, multiply by 0.357; creatinine to micromoles per liter, multiply by 88.4; and hematocrit to proportion of 1.0, multiply by 0.01.

^a^ Summary statistics reported among nonmissing values.

^b^ The enrollment priority groups reflect categories assigned to veterans on the basis of military service history, disability, income, Medicaid eligibility, and receipt of other VA benefits (eg, VA pension benefits). The 2 largest groups are: group 5, who are those without a service-connected disability but who have income below the VA health system adjusted income limit, are receiving VA pension benefits, or are eligible for Medicaid; and group 1, who typically have a service-connected disability rated 50% or more disabling or that prevents employment.

### Model Performance

The addition of socioeconomic characteristics to the CMS model modestly increased the C statistic from 0.77 (95% CI, 0.77-0.78) to 0.78 (95% CI, 0.78-0.78) for mortality after HF and from 0.73 (95% CI, 0.72-0.73) to 0.74 (95% CI, 0.73-0.74) for mortality after pneumonia (Table 2). The addition of socioeconomic factors to a model that includes CMS and clinical covariates did not increase the C statistic for HF (0.79 [95% CI, 0.79-0.79]) and yielded a modest increase in the C statistic for pneumonia (from 0.77 [95% CI, 0.77-0.78] to 0.78 [95% CI, 0.78-0.78]). Analyses restricted to veterans 66 years or older revealed similar findings (Table 2).

**Table 2.** Area Under Receiver Operating Curves for Mortality Models in Heart Failure and Pneumonia With or Without Adjustment for Socioeconomic Characteristics^a^

Characteristic	Heart failure		Pneumonia	
	CMS claims-based model (95% CI)	CMS claims-based model plus clinical covariates (95% CI)	CMS claims-based model (95% CI)	CMS claims-based model plus clinical covariates (95% CI)
Entire population				
Without socioeconomic factors	0.77 (0.77-0.78)	0.79 (0.79-0.79)	0.73 (0.72-0.73)	0.77 (0.77-0.78)
With socioeconomic factors	0.78 (0.78-0.78)	0.79 (0.79-0.79)	0.74 (0.73-0.74)	0.78 (0.78-0.78)
Age 66 y or older with Medicare enrollment				
Without socioeconomic factors	0.70 (0.70-0.70)	0.79 (0.79-0.79)	0.73 (0.73-0.73)	0.77 (0.77-0.78)
With socioeconomic factors	0.71 (0.70-0.71)	0.79 (0.79-0.79)	0.74 (0.74-0.74)	0.78 (0.78-0.78)

Abbreviation: CMS, Centers for Medicare & Medicaid Services.

^a^ Each value corresponds to the C statistic, a measure of the goodness of fit for binary outcomes in a logistic regression model. The C statistic is the area under the receiver operating curve.

### Change in Mortality Rates and Rankings After Adjustment for SES

**Figure 1.**
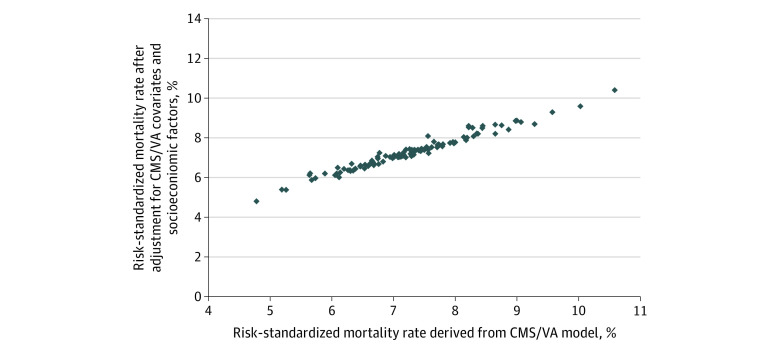
Veterans Affairs (VA) Medical Centers’ Claims-Based Risk-Standardized Mortality Rates Among Patients With Heart Failure With or Without Adjustment for Socioeconomic Factors Each point represents a VA Medical Center. The Spearman correlation between risk-standardized mortality rates with and without socioeconomic adjustment is 0.98. CMS indicates Centers for Medicare & Medicaid Services.

**Figure 2.**
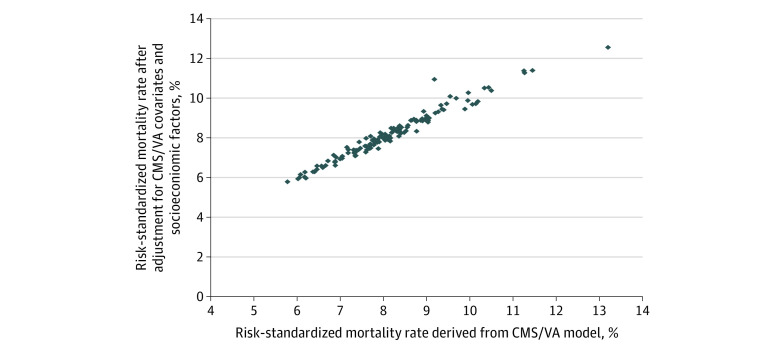
Veterans Affairs (VA) Medical Centers’ Claims-Based Risk-Standardized Mortality Rates Among Patients With Pneumonia With or Without Adjustment for Socioeconomic Factors Each point represents a VA Medical Center. The Spearman correlation between risk-standardized mortality rates with and without socioeconomic adjustment is 0.98. CMS indicates Centers for Medicare & Medicaid Services.

For both HF and pneumonia, the Spearman correlation in ranking between the CMS claims-based approach and an approach using the CMS claims and social risk factors was 0.98 (Figure 1 and Figure 2). Across all VAMCs, the mean (SD) absolute change in risk-adjusted mortality after adding social risk covariates was 0.11 (0.10) percentage points (IQR, 0.05-0.17 percentage points) for HF and 0.13 (0.13) percentage points (IQR, 0.04-0.18 percentage points) for pneumonia. We observed Spearman correlations of 0.98 to 0.99 in models that further included clinical covariates (eFigures 2 and 3 in the Supplement) and were restricted to veterans aged 66 years or older (eFigures 4 and 5 in the Supplement).

With the use of the CMS model for HF, the mean (SD) risk-standardized mortality rate of VAMCs during the period from 2012 to 2014 was 6.0% (0.4%) in the lowest quintile, 7.2% (0.4%) in the middle 3 quintiles, and 8.8% (0.6%) in the highest quintile (Table 3). After adding socioeconomic covariates, the mean (SD) risk-standardized mortality rate for these hospitals in 2012-2014 was 6.1% (0.4%) for those in the lowest quintile, 7.2% (0.4%) for those in the middle 3 quintiles, and 8.6% (0.5%) for those in the highest quintile. The mean absolute change in rank after socioeconomic adjustment was 3.0 ranking positions (IQR, 1.0-4.0) among hospitals in the highest quintile of mortality in HF and 4.4 ranking positions (IQR, 1.0-6.0) among VAMCs in the lowest quintile. Of the VAMCs in the highest quintile of 30-day mortality in HF using the CMS’s claims-based risk-adjustment model, none changed by more than 13 ranking positions after adding social risk factors to the risk-adjustment model. Overall, 16 hospitals (12.2%) moved across a quintile threshold after socioeconomic adjustment; 13 of these hospitals were in the middle 3 quintiles and the mean absolute change in risk-adjusted mortality was 0.08 percentage points (range, 0.02-0.13 percentage points) (Table 3).

**Table 3.** Risk-Standardized Mortality Rates and Changes in Rankings After Adjustment for Socioeconomic Factors Among VA Medical Centers in the Lowest, Middle 3, and Highest Quintiles of 30-Day Mortality Using the CMS and VA Health System Claims-Based Model

Characteristic	Lowest quintile	Middle 3 quintiles	Highest quintile
Heart failure			
30-d Risk-adjusted mortality, mean (SD), %			
CMS and VA health system claims-based model	6.0 (0.4)	7.2 (0.4)	8.8 (0.6)
CMS and VA health system claims-based model plus socioeconomic factors	6.1 (0.4)	7.2 (0.4)	8.6 (0.5)
VAMCs changing by >13 ranking positions, No./total No. (%)	2/26 (7.7)	7/79 (8.9)	0
Change in rank, mean (IQR)	0.8 (−2.0 to 3.0)	−0.2 (−4.0 to 4.0)	−0.3 (−3.0 to 2.0)
Absolute change in rank, mean (IQR)	4.4 (1.0 to 6.0)	5.7 (2.0 to 8.0)	3.0 (1.0 to 4.0)
VAMCs that change relative performance by ≥1, No./total No. (%)	2/26 (7.7)	13/79 (16.5)	1/26 (3.8)
Absolute change in risk-adjusted mortality, mean (range), %	0.13 (0.06 to 0.18)	0.08 (0.02 to 0.13)	0.18 (0.06 to 0.24)
Pneumonia			
30-d Risk-adjusted mortality, mean (SD), %			
CMS and VA health system claims-based model	6.6 (0.4)	8.1 (0.6)	10.0 (0.8)
CMS and VA health system claims-based model plus socioeconomic factors	6.6 (0.4)	8.1 (0.6)	10.0 (0.8)
VAMCs changing by >13 ranking positions, No./total No. (%)	1/26 (3.8)	7/78 (9.0)	1/26 (3.8)
Change in rank, mean (IQR)	1.3 (−1.0 to 2.0)	−0.1 (−6.0 to 5.0)	−1.0 (−3.0 to 1.0)
Absolute change in rank, mean (IQR)	2.7 (1.0 to 2.0)	6.3 (2.0 to 9.0)	3.4 (1.0 to 5.0)
VAMCs that change relative performance by ≥1 quintiles, No./total No. (%)	1/26 (3.8)	13/78 (16.7)	2/26 (7.7)
Absolute change in risk-adjusted mortality, mean (range), %	0.16 (0.10 to 0.21)	0.11 (0.03 to 0.16)	0.24 (0.17 to 0.33)

Abbreviations: CMS, Centers for Medicare & Medicaid Services; IQR, interquartile range; VA, Veterans Affairs; VAMC, Veterans Affairs Medical Center.

Using CMS’s model for pneumonia, the mean (SD) risk-standardized mortality rates for VAMCs was 6.6% (0.4%) in the lowest quintile, 8.1% (0.6%) for those in the middle 3 quintiles, and 10.0% (0.8%) for those in the highest quintile (Table 3). After adding socioeconomic covariates, the mean risk-standardized mortality rates for these hospitals were unchanged. Among VAMCs in the highest quintile for pneumonia mortality, 1 of 26 (3.8%) changed by more than 13 ranking positions. Sixteen VAMCs changed by 1 quintile after SES adjustment, with 13 of these hospitals in the middle 3 quintiles of mortality rates. Similar findings were observed for analyses that included clinical covariates (eTable 6 in the Supplement) and were limited to veterans aged 66 or older who were enrolled in Medicare (eTable 7 in the Supplement).

## Discussion

This study of changes in risk-adjusted mortality in VAMCs after adjusting for socioeconomic characteristics has 4 main findings. First, risk-adjustment models that included socioeconomic factors only minimally improved the discrimination of 30-day mortality when added to models that included CMS claims-based or novel clinical covariates. Second, the rankings of VAMCs with or without adjustment for socioeconomic characteristics were tightly correlated, with all correlation coefficients at or above 0.98. Third, few VAMCs with high mortality rates would be reclassified if socioeconomic characteristics were included in the current VA health system and CMS risk-adjustment model. For such facilities in the highest quintile of risk-standardized mortality rates (according to the CMS model), the mean absolute change in ranking position after adjusting for social risk factors was 3.0 for HF and 3.4 for pneumonia. Fourth, approximately 12% of VAMCs crossed a quintile threshold after the inclusion of socioeconomic covariates in the risk-adjustment model. The VAMCs that changed their quintile ranking were largely in the middle of the distribution of risk-adjusted mortality and had minimal absolute changes in adjusted mortality rates.

Our findings align with those of prior work showing that adjustment for social risk factors produced little change in hospitals’ rankings in readmission rates.^17,18,19,32^ Ross et al^33^ also reported that, among patients with Medicare fee-for-service coverage, safety-net hospitals had similar risk-adjusted mortality rates compared with non–safety-net hospitals. We extend this work in 3 main ways. First, we consider a broader range of social risk factors, including assessing neighborhood disadvantage at a more granular census block level rather than the 4-digit zip code or county level, using a composite of 17 measures of social risk.^34^ The use of a composite measure may capture social risk in a more comprehensive manner than the use of area-level income or poverty alone. Second, in contrast to studies of Medicare data that adjust for clinical risk using claims-based conditions, we evaluated the association of social risk factors in models that also included laboratory test values, vital signs, and ejection fraction, important factors associated with hospital mortality. Our study suggests that after including these novel clinical covariates in risk-adjustment models, adding social risk factor variables did not meaningfully change VAMCs’ risk-standardized mortality rates. Third, while other studies have focused on readmission, our analysis examined mortality, a widely reported hospital quality measure with evidence of disparities by SES.^35,36,37^ Prior work has found that hospitals’ risk-standardized mortality rates exhibit weak or no association with risk-standardized readmission rates.^38^

Our findings contrast with those of studies that report substantial changes in readmissions penalties and reduced variations in outcomes after adjusting for social risk factors.^16,20,21^ Our findings may have diverged from these prior studies for 3 reasons. First, because the VA health system prioritizes enrolling veterans with disabilities or who have low income, there may be more homogeneity in socioeconomic characteristics in the VA health system than in other health care systems. Prior studies have found that VA health system enrollees have lower income, less educational attainment, and worse health status, and are more likely to be a member of a racial/ethnic minority group than the general population.^39,40,41,42,43,44^ Second, other studies have reported lower magnitudes of disparity in care within the VA health system, including studies demonstrating that Black veterans have improved survival for some conditions compared with White veterans.^39,45,46^ This finding suggests that adjustments for social risk factors may have less association with changing assessments of VAMC outcomes compared with other health systems and payers. Third, it is possible that adjustment for social risk factors has less of an association with hospital mortality than with readmission.

The proliferation of public reporting and value-based payment initiatives has focused attention on the impact of these programs on hospitals that disproportionately serve patients with greater social disadvantage and the need to consider socioeconomic factors in measuring risk-adjusted performance rates.^12,13,14,15^ Failure to consider these factors may penalize hospitals based on the patients they serve rather than the quality of care they deliver, and therefore place further financial stress on safety-net hospitals and potentially widen disparities in care.^12,13,14^ However, some critics have expressed concern that adjustments for social risk factors may inappropriately set a lower standard of care in hospitals that serve socially disadvantaged patients and mask disparities by adjusting them away.^12,13,14^

Our study has 4 policy implications in the context of this ongoing debate. First, our study indicates that, at least in the case of hospital mortality measures for HF and pneumonia, adjusting for socioeconomic factors did not yield substantial changes in adjusted mortality rates for VAMCs. Second, even with minimal absolute changes in risk-adjusted performance, approximately 12% of VAMCs crossed a quintile threshold after inclusion of socioeconomic covariates. Thus, inclusion of socioeconomic or other covariates can have material consequences for some hospitals if policy makers use strict thresholds (such as being on either side of a quintile) to publicly report and reward performance. Third, our study demonstrates the feasibility of including socioeconomic factors in risk-adjustment models to gauge the association of these adjustments with assessments of hospital performance. Policy makers and other stakeholders in the VA health system could consider applying similar approaches to other outcome measures. Fourth, health systems can measure and evaluate performance with or without adjustment for social risk factors and stratify performance rates for disadvantaged populations so that disparities remain visible.^47^

### Limitations

Our study has some limitations. First, our study is limited to 2 hospital performance measures and may not be generalizeable to other quality indicators. Mortality after admission for HF and pneumonia has been a focus of public reporting and pay-for-performance initiatives for more than a decade; there may be reduced variation on these indicators as hospitals have learned how to optimize performance. Furthermore, the measures in this study examined death after admission; the findings may differ for outcomes after discharge, including readmission. Second, our findings are limited to the VA health care system and may not extend to other health care settings. Third, SES is a multidimensional construct, and we lacked other individual-level data on important socioeconomic factors, such as educational level, health literacy, and social support. However, our study included more detailed measures than previous studies, and neighborhood-level disadvantage is an important contextual variable in assessing hospital outcomes.^48^

## Conclusions

Risk adjustment for socioeconomic factors did not meaningfully change VAMCs’ performance on mortality measures in patients with HF and pneumonia. The implications of such adjustments should be examined for other quality measures and health systems.

## Notes

Supplement. **eTable 1.** ICD-9 Codes for Primary Diagnoses **eTable 2.** Prevalence of Claims-Based Comorbidities of Veterans Admitted with Heart Failure and Pneumonia, 2012-2014 **eTable 3.** Summary of Missing Data for Clinical and Socioeconomic Covariates **eTable 4.** VA Medical Center–Level Variations in Socioeconomic Characteristics **eTable 5.** Unadjusted 30-Day Mortality Rates, by Selected Socioeconomic Characteristics **eTable 6.** Changes in Mortality Rates and Rankings After Adding Socioeconomic Factors to Risk Adjustment Models With Claims-Based and Clinical Covariates, by Quintile of VA Medical Centers’ Mortality Rates **eTable 7.** Changes in Mortality Rates and Rankings for Patients Age 66 and Older After Adding Socioeconomic Factors to Risk Adjustment Models With Claims-Based Covariates, by Quintile of VA Medical Centers’ Mortality Rate **eFigure 1.** Flowchart for the Study Population **eFigure 2.** VA Medical Centers’ Risk-Standardized Mortality Rates in Heart Failure With and Without Including Socioeconomic Factors in Models Adjusting for Claims-Based and Clinical Covariates **eFigure 3.** VA Medical Centers’ Risk-Standardized Mortality Rates in Pneumonia With and Without Including Socioeconomic Factors in Models Adjusting for Claims-Based and Clinical Covariates **eFigure 4.** VA Medical Centers’ Risk-Standardized Mortality Rates in Heart Failure With and Without Including Socioeconomic Factors Among Patients Age 66 and Older **eFigure 5.** VA Medical Centers’ Risk-Standardized Mortality Rates in Pneumonia With and Without Including Socioeconomic Factors Among Patients Age 66 and Older
