# Supplementary material for: Evaluation of Changes in Veterans Affairs Medical Centers’ Mortality Rates After Risk Adjustment for Socioeconomic Status
Source: JAMA Netw Open. 2020 Dec 3;3(12):e2024345. doi: 10.1001/jamanetworkopen.2020.24345 (PMC7716194; doi:10.1001/jamanetworkopen.2020.24345)
Supplement: Supplement. — eTable 1. ICD-9 Codes for Primary Diagnoses eTable 2. Prevalence of Claims-Based Comorbidities of Veterans Admitted with Heart Failure and Pneumonia, 2012-2014 eTable 3. Summary of Missing Data for Clinical and Socioeconomic Covariates eTable 4. VA Medical Center–Level Variations in Socioeconomic Characteristics eTable 5. Unadjusted 30-Day Mortality Rates, by Selected Socioeconomic Characteristics eTable 6. Changes in Mortality Rates and Rankings After Adding Socioeconomic Factors to Risk Adjustment Models With Claims-Based and Clinical Covariates, by Quintile of VA Medical Centers’ Mortality Rates eTable 7. Changes in Mortality Rates and Rankings for Patients Age 66 and Older After Adding Socioeconomic Factors to Risk Adjustment Models With Claims-Based Covariates, by Quintile of VA Medical Centers’ Mortality Rate eFigure 1. Flowchart for the Study Population eFigure 2. VA Medical Centers’ Risk-Standardized Mortality Rates in Heart Failure With and Without Including Socioeconomic Factors in Models Adjusting for Claims-Based and Clinical Covariates eFigure 3. VA Medical Centers’ Risk-Standardized Mortality Rates in Pneumonia With and Without Including Socioeconomic Factors in Models Adjusting for Claims-Based and Clinical Covariates eFigure 4. VA Medical Centers’ Risk-Standardized Mortality Rates in Heart Failure With and Without Including Socioeconomic Factors Among Patients Age 66 and Older eFigure 5. VA Medical Centers’ Risk-Standardized Mortality Rates in Pneumonia With and Without Including Socioeconomic Factors Among Patients Age 66 and Older [file jamanetwopen-e2024345-s001.pdf]

Supplementary Online Content

Trivedi AN, Jiang L, Silva G, et al. Evaluation of changes in Veterans Affairs medical centers' mortality rates after risk adjustment for socioeconomic status. *JAMA Netw Open.* 2020;3(12):e2024345. doi:10.1001/jamanetworkopen.2020.24345

eTable 1. ICD-9 Codes for Primary Diagnoses
eTable 2. Prevalence of Claims-Based Comorbidities of Veterans Admitted with Heart Failure and Pneumonia, 2012-2014
eTable 3. Summary of Missing Data for Clinical and Socioeconomic Covariates
eTable 4. VA Medical Center–Level Variations in Socioeconomic Characteristics
eTable 5. Unadjusted 30-Day Mortality Rates, by Selected Socioeconomic Characteristics
eTable 6. Changes in Mortality Rates and Rankings After Adding Socioeconomic Factors to Risk Adjustment Models With Claims-Based and Clinical Covariates, by Quintile of VA Medical Centers' Mortality Rates
eTable 7. Changes in Mortality Rates and Rankings for Patients Age 66 and Older After Adding Socioeconomic Factors to Risk Adjustment Models With Claims-Based Covariates, by Quintile of VA Medical Centers' Mortality Rate
eFigure 1. Flowchart for the Study Population
eFigure 2. VA Medical Centers' Risk-Standardized Mortality Rates in Heart Failure With and Without Including Socioeconomic Factors in Models Adjusting for Claims-Based and Clinical Covariates
eFigure 3. VA Medical Centers' Risk-Standardized Mortality Rates in Pneumonia With and Without Including Socioeconomic Factors in Models Adjusting for Claims-Based and Clinical Covariates
eFigure 4. VA Medical Centers' Risk-Standardized Mortality Rates in Heart Failure With and Without Including Socioeconomic Factors Among Patients Age 66 and Older
eFigure 5. VA Medical Centers' Risk-Standardized Mortality Rates in Pneumonia With and Without Including Socioeconomic Factors Among Patients Age 66 and Older

This supplementary material has been provided by the authors to give readers additional information about their work.

© 2020 Trivedi AN et al. *JAMA Network Open.*

eTable 1. ICD-9 Codes for Primary Diagnoses

Primary Diagnoses	ICD-9 Codes/Descriptions
Heart Failure	402.01, 402.11, 402.91, 404.01, 404.03, 404.11, 404.13, 404.91, 404.93, 428.0, 428.1, 428.20, 428.21, 428.22, 428.23, 428.30, 428.31, 428.32, 428.33, 428.40, 428.41, 428.42, 428.43, and 428.9
Pneumonia	480.0, 480.1, 480.2, 480.3, 480.8, 480.9, 481, 482.0, 482.1, 482.2, 482.30, 482.31, 482.32, 482.39, 482.40, 482.41, 482.42, 482.49, 482.81, 482.82, 482.83, 482.84, 482.89, 482.9, 483.0, 483.1, 483.8, 485, 486, 487.0, and 488.11

© 2020 Trivedi AN et al. *JAMA Network Open.*

eTable 2. Prevalence of Claims-Based Comorbidities of Veterans Admitted with Heart Failure and Pneumonia, 2012-2014

Characteristic		Heart Failure N=42892	Pneumonia N=39062
CMS Claims-Based Conditions	Percutaneous Transluminal Coronary Angioplasty	9.51	4.97
	Coronary Artery Bypass Graft	17.87	7.66
	Congestive Heart Failure	73.35	26.6
	Acute Myocardial Infarction	7.05	2.49
	Ischemic Heart Disease	10.33	4.24
	Chronic Atherosclerosis	60.98	35.2
	Cardiorespiratory Failure and Shock	17.03	15.45
	Rheumatic	19.64	
	Hypertension	87.45	74.89
	Stroke	9.92	8.72
	Cerebrovascular		10.39
	Renal Failure	47.15	27.92
	Chronic Obstructive Pulmonary Disease	40.79	47.28
	Pneumonia	19.99	34.74
	DM	59.15	
	Malnutrition		4.35
	Dementia/Senility	11	15.14
	Functional Disability	8.44	8.44
	Peripheral Vascular Disease	28.58	21.77
	Cancer	4.06	11.56
	Trauma	26.98	28.06
	Major Psychiatric Disease	13.91	19.13
	Chronic Liver Disease	6.04	5.98
	Hematological Disorder		1.66
	Iron Deficiency		34.48
	Depression		24.89
	Parkinson’s Disease		2.28
	Seizure Disorder		4.46
	Lung Fibrosis		6.75
	Asthma		5.64
	Vertebral Fracture		1.2

© 2020 Trivedi AN et al. *JAMA Network Open.*

eTable 3. Summary of Missing Data for Clinical and Socioeconomic Covariates

Variable	Heart Failure (n=42,892)		Pneumonia (n=39,062)	
	Number Missing	%	Number Missing	%
Systolic Blood Pressure	372	0.87	298	0.76
Diastolic Blood Pressure	386	0.90	294	0.75
Heart Rate	401	0.93	327	0.84
Respiratory Rate	436	1.02	345	0.88
Pulse Oximetry	1,186	2.77	902	2.31
Body Mass Index	2,628	6.13	2,822	7.22
Sodium	1,410	3.29	1,232	3.15
Potassium	1,674	3.90	1,504	3.85
Blood Urea Nitrogen	2,806	6.54	2,472	6.33
Creatinine	1,122	2.62	874	2.24
Hematocrit	1,498	3.49	1,478	3.78
B-type Natriuretic Peptide	7885	18.38	-	-
Ejection Fraction	10184	23.74	-	-
Area Deprivation Index	395	0.92	424	1.09
Race/Ethnicity	222	0.52	250	0.64

© 2020 Trivedi AN et al. *JAMA Network Open.*

eTable 4. VA Medical Center–Level Variations in Socioeconomic Characteristics

Hospital Level	CHF				Pneumonia			
N=131	Mean	Median	25th percentile	75th percentile	Mean	Median	25th percentile	75th percentile
% Black	19.68	12.98	4.26	26.87	14.88	9.66	3.56	20.33
% Hispanic	2.07	1.49	0.82	2.50	2.26	1.74	0.92	2.80
Priority Categories								
Group 1	23.78	23.51	20.41	27.04	27.59	26.75	23.12	30.58
Group 2	5.46	5.47	4.42	6.52	6.07	5.95	4.76	7.01
Group 3	9.11	8.88	7.27	10.33	9.41	9.44	7.73	10.79
Group 4	5.24	4.75	3.43	6.11	5.49	5.37	3.53	6.76
Group 5	49.58	50.16	44.33	53.11	45.08	44.72	40.66	50.26
Group 6	0.70	0.62	0.26	0.98	1.05	0.88	0.40	1.42
Group 7	5.81	4.48	3.36	6.71	5.00	4.21	3.03	6.25
Group 8	0.31	0	0	0.46	0.31	0.25	0	0.44
ADI	105.41	107.79	102.54	110.51	104.92	107.72	102.18	110.11
Nursing home use in year prior to admission, %	14.88	13.77	11.17	16.83	13.59	12.68	9.92	16.47
Direct admission from nursing home, %	3.01	2.15	1.11	3.26	3.68	3.21	1.73	5.07
Rural, %	40.98	37.99	19.01	56.56	39.63	35.91	17.01	55.65
Homeless, %	10.49	9.23	6.04	13.28	12.35	11.38	7.48	16.13

© 2020 Trivedi AN et al. *JAMA Network Open.*

eTable 5. Unadjusted 30-Day Mortality Rates, by Selected Socioeconomic Characteristics

	CHF	Pneumonia
Overall	3086 (7.19)	3153 (8.07)
Race/Ethnicity, n (%)		
White	2304 (7.80)	2453 (8.27)
Black	493 (4.94)	431 (7.10)
Hispanic	67 (7.52)	65 (7.51)
Priority Categories, n (%)		
Group 1	662(6.15)	790 (7.34)
Group 2	168(7.02)	156 (6.68)
Group 3	323(8.23)	302 (8.19)
Group 4	198(8.95)	219 (9.30)
Group 5	1533(7.23)	1472 (8.34)
Group 6	22(7.07)	28 (7.57)
Group 7/8	180(8.54)	186 (9.74)
Area Deprivation Index, n (%)		
Lowest Quintile	547 (6.44)	614(7.95)
Middle Three Quintiles	1872 (7.34)	1864(8.04)
Highest Quintile	639 (7.52)	639(8.27)
Nursing home use in year prior to admission, n (%)		
Yes	724(12.50)	840 (15.71)
No	2362 (6.36)	2313 (6.86)
Direct admission from nursing home, n (%)		
Yes	195(19.74)	353 (22.82)
No	2891(6.90)	2800(7.46)
Rural, n (%)		
Yes	1045(7.45)	1135(8.11)
No	2041(7.07)	2018(8.05)
Homeless, n (%)		
Yes	386 (8.06)	434(8.79)
No	2700 (7.09)	2719(7.97)

© 2020 Trivedi AN et al. *JAMA Network Open.*

eTable 6. Changes in Mortality Rates and Rankings After Adding Socioeconomic Factors to Risk Adjustment Models With Claims-Based and Clinical Covariates, by Quintile of VA Medical Centers' Mortality Rates

	Lowest Quintile	Middle Three Quintiles	Highest Quintile
**Heart Failure**			
30-day Risk-Adjusted Mortality (CMS/VA Claims-based Model plus clinical variables), %	6.0%	7.2%	8.7%
30-day Risk-Adjusted Mortality (CMS/VA Claims-based Model plus clinical variables plus socioeconomic factors), %	6.1%	7.2%	8.6%
VAMCs Changing by >13 ranking positions	0 (0%)	3 (3.8%)	0 (0%)
Mean Change in Rank (IQR)	0.5 (-2, 5)	-0.1 (-3, 3)	-0.2 (-2, 2)
Mean Absolute Change in Rank (IQR)	2.9 (1, 5)	4.2 (1, 7)	2.4 (1, 3)
**Pneumonia**			
30-day Risk-Adjusted Mortality (CMS/VA Claims-based Model plus clinical variables), %	6.6%	8.1%	10.0%
30-day Risk-Adjusted Mortality (CMS/VA Claims-based Model plus clinical variables plus socioeconomic factors), %	6.6%	8.1%	10.12%
VAMCs Changing by >13 ranking positions	0 (0%)	3 (3.80%)	0 (0%)
Mean Change in Rank (IQR)	0.2 (-2, 0)	0.1 (-4, 3)	-0.4 (-2, 1)
Mean Absolute Change in Rank (IQR)	1.5 (0, 2)	4.6 (2, 7)	2.5 (1, 3)

© 2020 Trivedi AN et al. *JAMA Network Open.*

eTable 7. Changes in Mortality Rates and Rankings for Patients Age 66 and Older After Adding Socioeconomic Factors to Risk Adjustment Models With Claims-Based Covariates, by Quintile of VA Medical Centers' Mortality Rate

	Lowest Quintile	Middle Three Quintiles	Highest Quintile
**Heart Failure**			
30-day Risk-Adjusted Mortality (CMS/VA Claims-based Model), %	7.2	8.7	10.5
30-day Risk-Adjusted Mortality (CMS/VA Claims-based Model plus socioeconomic factors), %	7.4	8.7	10.2
VAMCs Changing by >13 ranking positions	3 (11.5%)	7 (8.9%)	1 (3.9%)
Mean Change in Rank (IQR)	2.5<br>(-1, 3)	-0.5<br>(-6, 4)	-0.8<br>(-2, 1)
Mean Absolute Change in Rank (IQR)	4.8<br>(1, 4)	6.2<br>(2, 9)	3.7<br>(0, 5)
**Pneumonia**			
30-day Risk-Adjusted Mortality (CMS/VA Claims-based Model), %	8.3	9.9	12.2
30-day Risk-Adjusted Mortality (CMS/VA Claims-based Model plus socioeconomic factors), %	8.4	9.9	12.3
VAMCs Changing by >13 ranking positions	1 (3.9%)	11 (14.1%)	1 (3.9%)
Mean Change in Rank (IQR)	0.6<br>(-2, 2)	0.03<br>(-5, 6)	-0.7<br>(-3, 1)
Mean Absolute Change in Rank (IQR)	2.7<br>(1, 3)	6.6<br>(2, 9)	4.3<br>(1, 8)

© 2020 Trivedi AN et al. *JAMA Network Open.*

eFigure 1. Flowchart for the Study Population

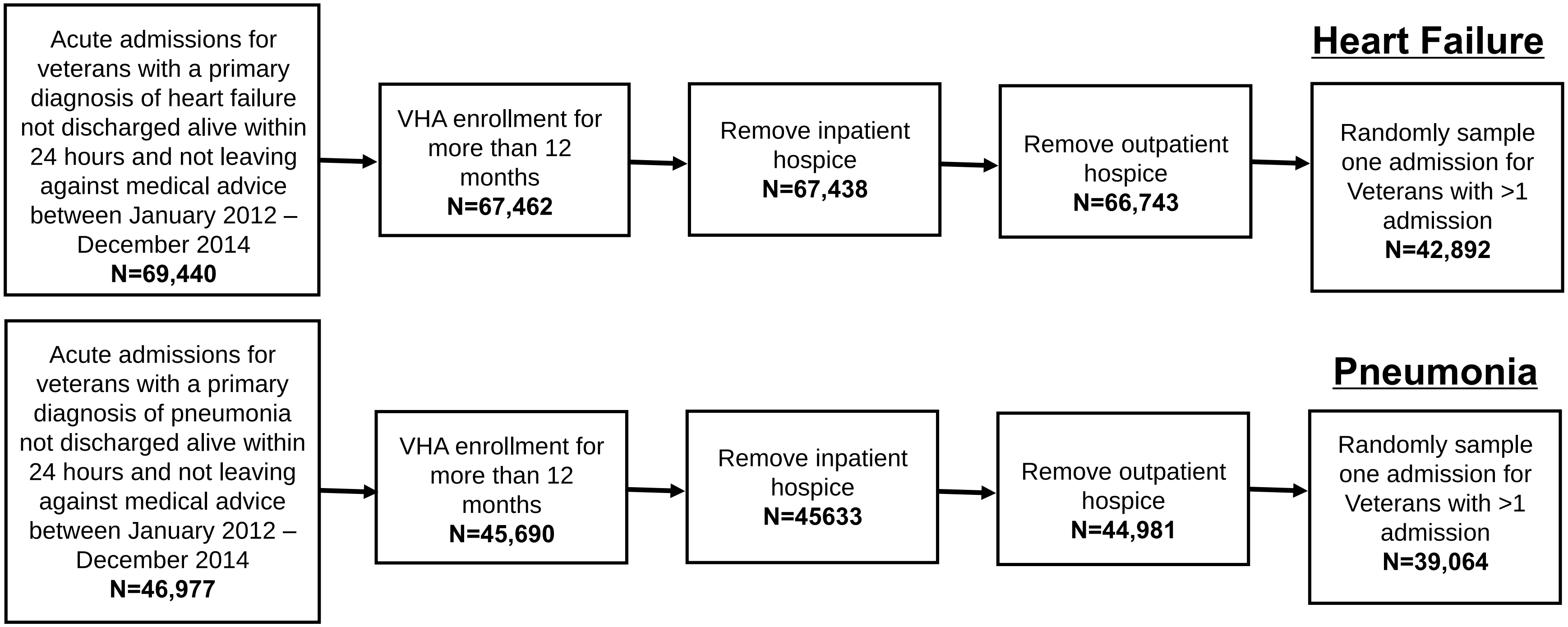

© 2020 Trivedi AN et al. *JAMA Network Open*.

eFigure 2. VA Medical Centers' Risk-Standardized Mortality Rates in Heart Failure With and Without Including Socioeconomic Factors in Models Adjusting for Claims-Based and Clinical Covariates

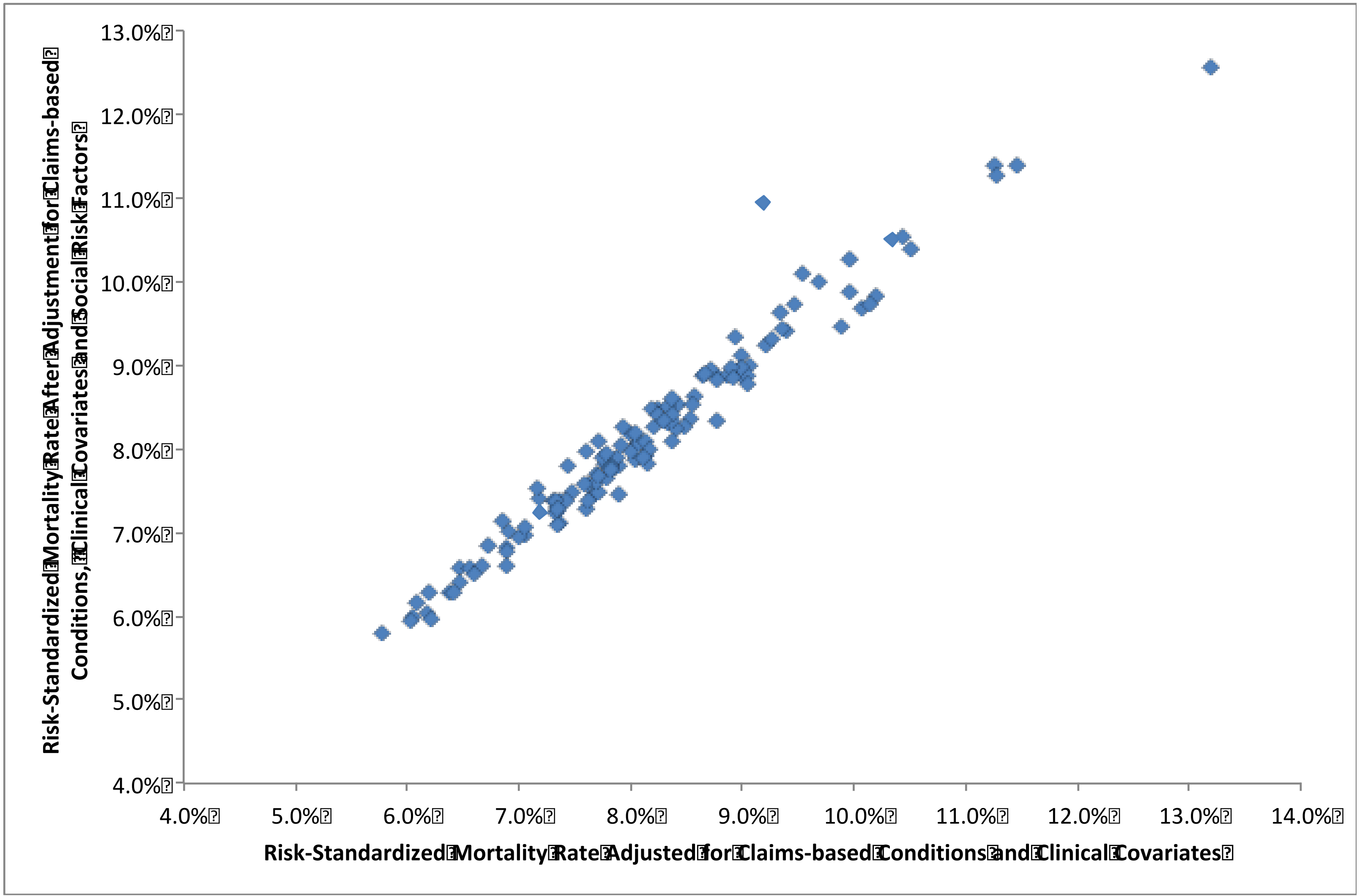

Notes: Each point represents a VA Medical Center. The Spearman's correlation between risk-standardized mortality rates with and without socioeconomic adjustment is 0.99.

© 2020 Trivedi AN et al. *JAMA Network Open.*

eFigure 3. VA Medical Centers' Risk-Standardized Mortality Rates in Pneumonia With and Without Including Socioeconomic Factors in Models Adjusting for Claims-Based and Clinical Covariates

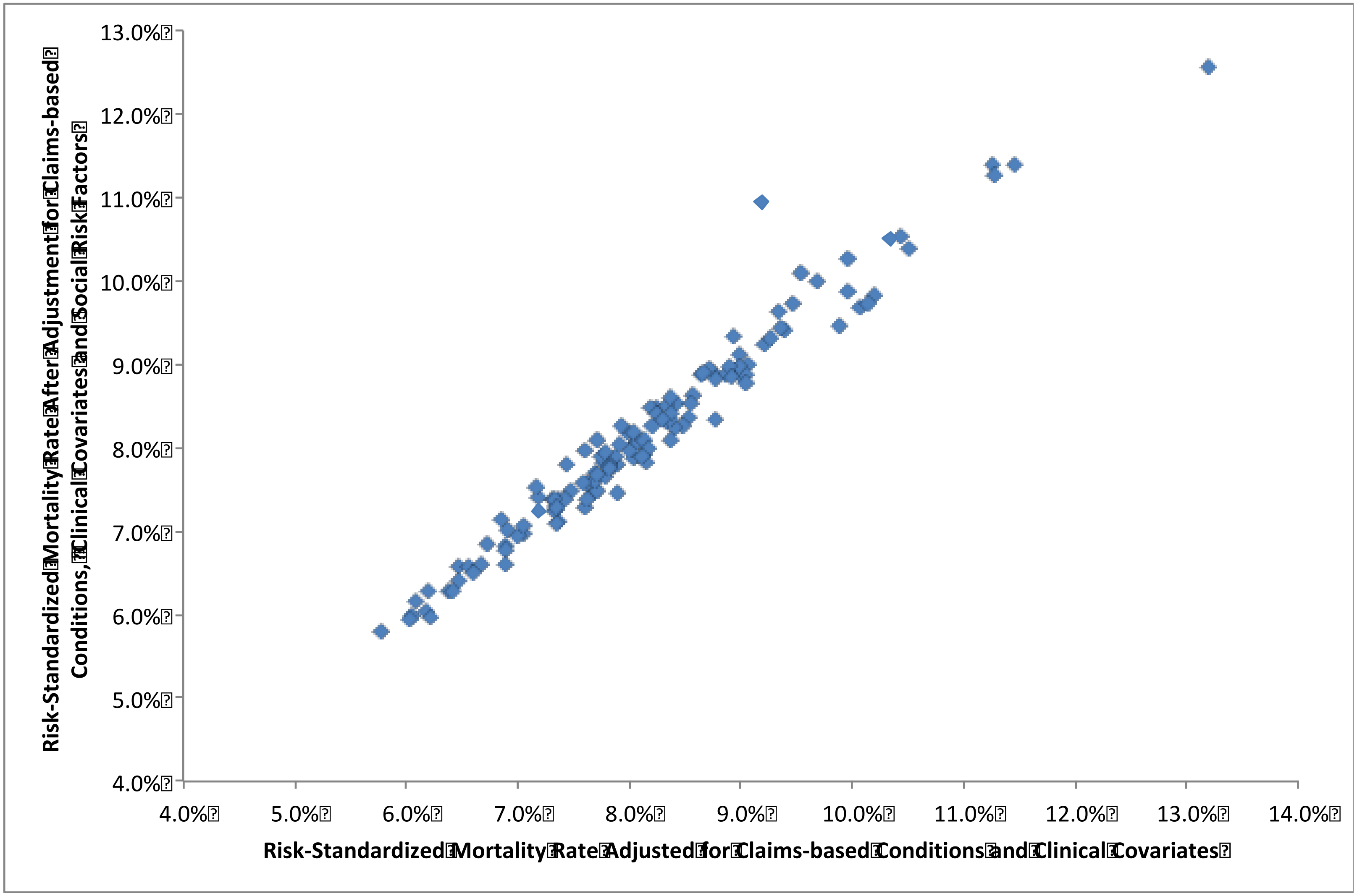

Notes: Each point represents a VA Medical Center. The Spearman's correlation between risk-standardized mortality rates with and without socioeconomic adjustment is 0.98.

© 2020 Trivedi AN et al. *JAMA Network Open.*

eFigure 4. VA Medical Centers' Risk-Standardized Mortality Rates in Heart Failure With and Without Including Socioeconomic Factors Among Patients Age 66 and Older

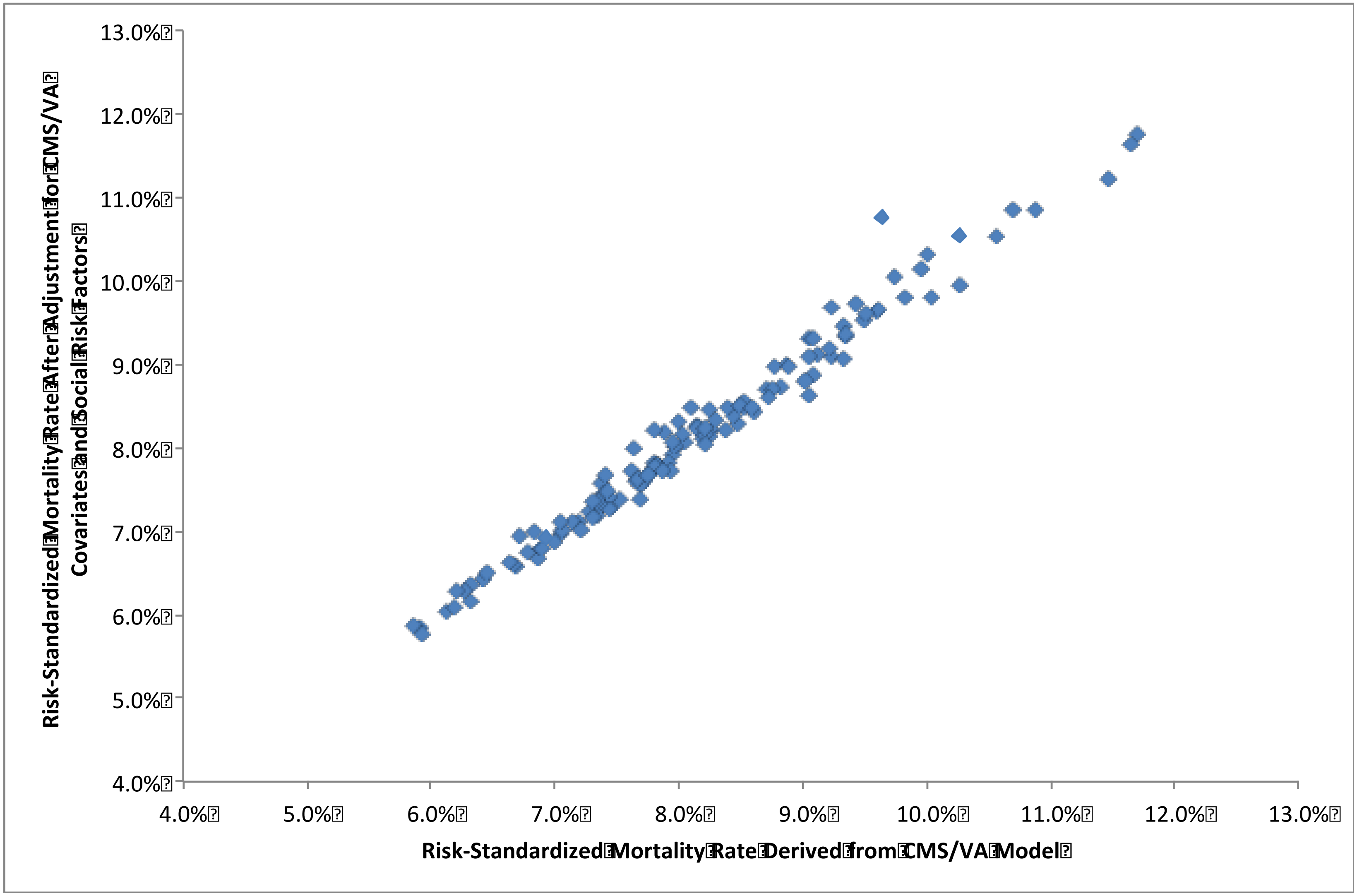

Notes: Each point represents a VA Medical Center. The Spearman's correlation between risk-standardized mortality rates with and without socioeconomic adjustment is 0.98.

© 2020 Trivedi AN et al. *JAMA Network Open.*

eFigure 5. VA Medical Centers' Risk-Standardized Mortality Rates in Pneumonia With and Without Including Socioeconomic Factors Among Patients Age 66 and Older

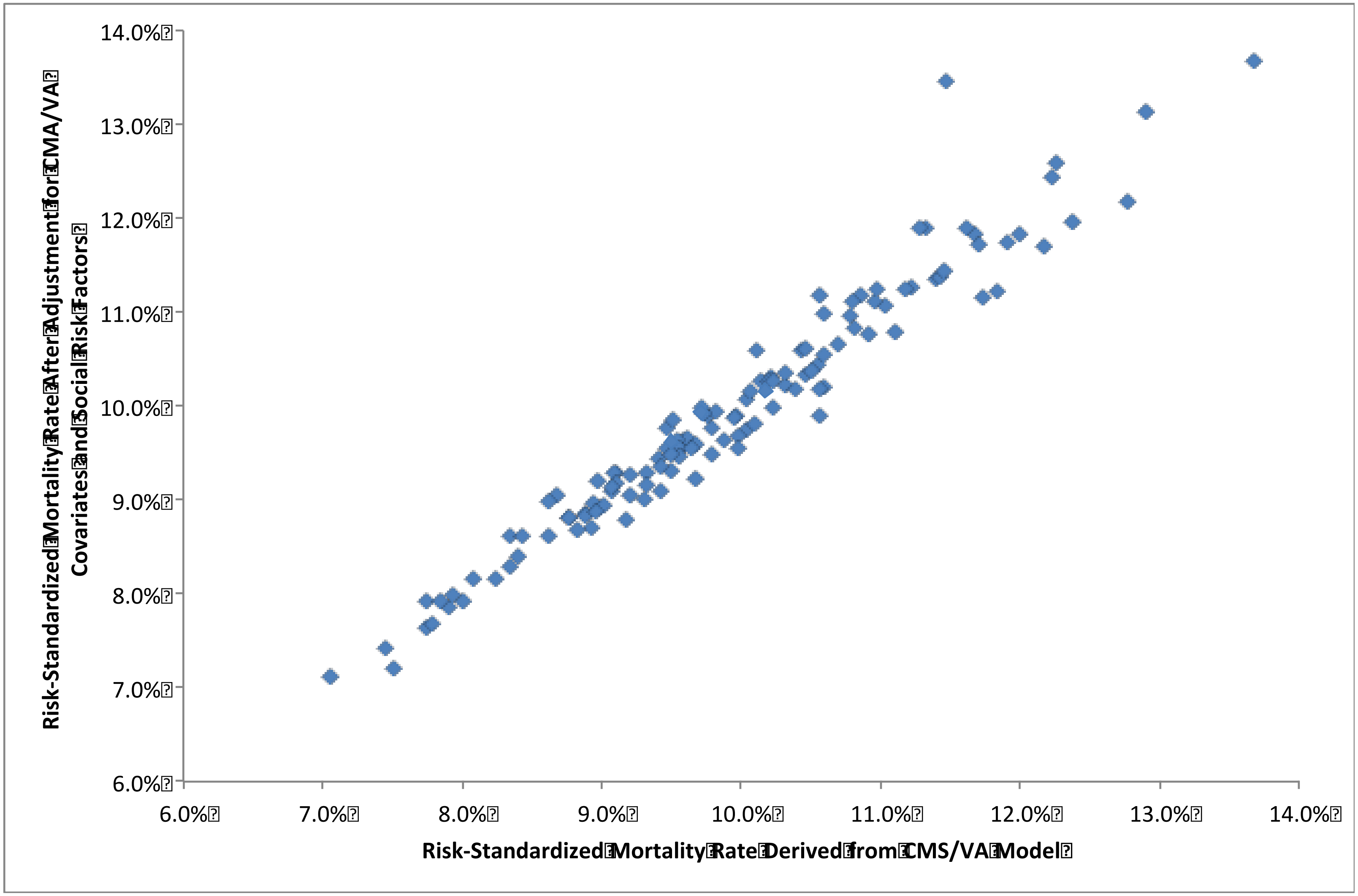

Notes: Each point represents a VA Medical Center. The Spearman's correlation between risk-standardized mortality rates with and without socioeconomic adjustment is 0.98.

© 2020 Trivedi AN et al. *JAMA Network Open.*
